# Directional genetic differentiation and relative migration

**DOI:** 10.1002/ece3.2096

**Published:** 2016-04-20

**Authors:** Lisa Sundqvist, Kevin Keenan, Martin Zackrisson, Paulo Prodöhl, David Kleinhans

**Affiliations:** ^1^ Department of Marine Sciences University of Gothenburg SE‐405 30 Gothenburg Sweden; ^2^ School of Biological Sciences Institute for Global Food Security Queen's University Belfast Belfast BT9 7BL UK; ^3^ Department for Chemistry and Molecular Biology University of Gothenburg SE‐405 30 Gothenburg Sweden; ^4^ ForWind Center for Wind Energy Research Institute of Physics Carl von Ossietzky University DE‐26129 Oldenburg Germany

**Keywords:** Allele frequency data, asymmetric migration, directional gene flow, dispersal

## Abstract

Understanding the population structure and patterns of gene flow within species is of fundamental importance to the study of evolution. In the fields of population and evolutionary genetics, measures of genetic differentiation are commonly used to gather this information. One potential caveat is that these measures assume gene flow to be symmetric. However, asymmetric gene flow is common in nature, especially in systems driven by physical processes such as wind or water currents. As information about levels of asymmetric gene flow among populations is essential for the correct interpretation of the distribution of contemporary genetic diversity within species, this should not be overlooked. To obtain information on asymmetric migration patterns from genetic data, complex models based on maximum‐likelihood or Bayesian approaches generally need to be employed, often at great computational cost. Here, a new simpler and more efficient approach for understanding gene flow patterns is presented. This approach allows the estimation of directional components of genetic divergence between pairs of populations at low computational effort, using any of the classical or modern measures of genetic differentiation. These directional measures of genetic differentiation can further be used to calculate directional relative migration and to detect asymmetries in gene flow patterns. This can be done in a user‐friendly web application called divMigrate‐online introduced in this study. Using simulated data sets with known gene flow regimes, we demonstrate that the method is capable of resolving complex migration patterns under a range of study designs.

## Introduction

Measures of population genetic differentiation are widely used in studies focusing on conservation, management, and evolution. Generally, information about the pattern of population structure and the level of differentiation is derived from allele frequency data. The most commonly used measures, utilizing this information, being Wright's fixation index $F_{\mathrm{st}}$ (Wright 1943), Nei's $G_{\mathrm{st}}$ (Nei 1973), and the more recently introduced $G_{\mathrm{st}}^{\prime }$ (Hedrick and Goodnight 2005) and *D* (Jost 2008), which are independent of gene diversity.

A particularly useful feature of these parameters is that, assuming an island model of population structure, they can be used to estimate migration among populations (Wright 1931, 1949; Jost 2008). These measures, however, assumes migration to be symmetric (i.e., of equal rate in all directions). For simplification, the term migration is here and for the rest of this study used interchangeably with the term gene flow.

Structured populations characterized by asymmetric migration is, on the other hand, common in nature, in particular in systems driven by physical transport processes, such as wind or water currents (Pringle et al. 2011), or those with strong habitat quality gradients, which can lead to competition‐driven directional dispersal (Paz‐Vinas et al. 2013). In marine environments, ocean currents are known to affect both the dispersal of planktonic and benthic species, the latter often being characterized with a planktonic phase during early stages of development (Siegel et al. 2003; Cowen and Sponaugle 2009). Other examples, where the direction of gene flow can be affected by physical processes, include organism living in river systems (e.g., impassable waterfalls prevent upstream dispersal), as well as wind‐pollinated plants, mosses, and lichen (Munõz et al., 2004; Hänfling and Weetman, 2006; Friedman and Barrett 2009).

Both physical processes and density‐dependent competition can lead to source–sink dynamics among populations (i.e., metapopulation structure). Where this occurs, parameters such as genetic differentiation can be significantly skewed (Dias 1996). This, in turn, can lead to incorrect conservation and/or management decisions. For instance, while sink populations of poor quality (i.e., low/no intrapopulation recruitment) contribute little to the long‐term evolutionary potential of the species (Whitlock and McCauley 1999), they can display greater genetic differentiation relative to source populations (Whitlock and McCauley 1999). As a consequence, they can be incorrectly regarded as a “unique" component of the species’ overall diversity, which may misdirect conservation efforts.

In asymmetric systems, it becomes important to estimate directional migration to fully understand the processes leading to genetic structuring of populations, as well as to allow for effective conservation and management decisions to be derived from such information (e.g., Pringle et al. 2011). For example, when aiming to conserve endangered species, implementing management strategies for source populations is known to be more effective than for sink populations (Dias 1996). In addition to the difficulties associated with understanding genetic structure, the absence of information about the symmetry of gene flow can also make the inference of past demographic changes within populations more difficult. For instance, Paz‐Vinas et al. (2013) recently demonstrated, using both simulated and empirical data, that the probability of incorrectly inferring past population expansions increased dramatically when populations were characterized by asymmetric patterns of gene flow. Thus, the ability to detect asymmetric migration, when it is present, is essential for the understanding of evolutionary processes that have led to contemporary diversity patterns.

Currently, to obtain information about patterns of migration from genetic data in asymmetric systems, complex mathematical models using maximum‐likelihood or Bayesian approaches are typically required (e.g.,Wilson and Rannala 2003; Beerli 2009). In these models, a large number of parameters are estimated simultaneously involving complex optimization algorithms, resulting in intensive computational requirements. As a consequence, these models are often used as *black boxes* implying that users, due to the complexity of these analytical approaches, typically only have a limited understanding of the underlying models and their assumptions. Thus, most users are not in a position to adequately assess when their application is inappropriate.

In contrast, this study introduces a new, relatively simple and tangible method for the detection of asymmetric migration from allele frequency data. This approach provides robust information on the direction of migration and is intended to fill the gap between existing complex methods for measuring asymmetric migration and symmetric measures of genetic differentiation. The method is based on defining a *hypothetical pool of migrants* for a given pair of populations and estimating an appropriate measure of genetic differentiation between each of the two populations and the hypothetical pool. The directional genetic differentiation can then be used to estimate the relative levels of migration between the two populations. The larger of the two relative migration values indicates the population that is likely to behave as a source population (i.e., the hypothetical pool of migrants is genetically more similar to this population), while the smaller of the two estimates indicates the population most likely to behave as a sink. By testing whether these two genetic distances are significantly different from one another, it is also possible to determine whether migration occurs at a significantly higher rate in one direction over the other. The concept of a hypothetical pool of migrants makes it possible to gain new information about direction of gene flow using regular symmetric measures of genetic differentiation.

This new approach opens up a new dimension of applications where directional measures of genetic differentiation can be used both to explore and to detect asymmetric migration patterns. It is argued that this tool will be of major utility in molecular ecological studies where information about the presence/absence of genetic connectivity and its dynamics among populations is of interest. Consider, for example, the benefit of being able to explicitly identify source and sink populations in metapopulations, having the ability to test whether physical barriers precluded gene flow in a particular direction, or the ability to understand the influence of ecosystem processes on gene flow (e.g., is the direction of migration among populations correlated with an ocean current). Accordingly, we present this method as a new tool to researchers interested in a better understanding of the demographic and evolutionary dynamics of populations and species and more specifically on the use of this information for conservation and management.

## Theory

### Measures of genetic differentiation

For measures of genetic differentiation, a value of zero indicates that the allele frequencies among populations are equal, while values larger than zero represent increasing differences (Meirmans and Hedrick 2011). Generally, these genetic differentiation measures are based on two parameters, which describe the distribution of genetic diversity among populations, namely the mean heterozygosity in the total population ($H_{t}$) and the mean heterozygosity within the individual populations ($H_{s}$) (Meirmans and Hedrick 2011). While an extensive formal set of notations exists for these two parameters, as well as for $G_{\mathrm{st}}$ and *D*, in here, we introduce vector notations which make it possible to carry out calculations for a number of populations simultaneously.

Let the total number of different alleles present in *P* populations be *N*. For now, equal population sizes are assumed. The allele frequencies in the individual populations can then be arranged in the *N* × *P*‐matrix *A*.

(1) $$A=\left(\begin{array}{ccc} a_{11} & \cdots & a_{1P}\\ \vdots & \cdots & \vdots \\ a_{N1} & \cdots & a_{NP} \end{array}\right)=\left(a_{1},\cdots ,a_{P}\right),$$

where the matrix element $a_{ij}$ represents the frequency of allele *i* in population *j* with $\sum_{i}a_{ij}\;=\;1$ for any population *j*. The column vectors $a_{j}\in {\left[0,1\right]}^{N}$ constitute the allele frequencies in the individual populations, please note that bold letters indicate vectors. For each population, the degree of heterozygosity (*H*) can be estimated from the vector of allele frequencies $a\in {\left[0,1\right]}^{N}$ as follows:

(2) $$H\left(a\right)=1-a^{T}a=1-{\left|a\right|}^{2}.$$

For a pairwise comparison involving two populations with allele frequencies $a\in {\left[0,1\right]}^{N}$ and $b\in {\left[0,1\right]}^{N}$, within‐ population heterozygosity ($H_{s}$) and total‐population heterozygosity ($H_{t}$) can be estimated from equation (2) as follows:

(3a) $$H_{t}\left(a,b\right)=1-\frac{1}{4}{\left|a+b\right|}^{2},$$

(3b) $$H_{s}\left(a,b\right)=1-\frac{1}{2}{\left(|a\right|}^{2}+{\left|b\right|}^{2}).$$

From these expressions, measures for genetic differentiation between populations with allele frequencies ***a*** and ***b*** can be defined using vector algebra:

(4a) $$D_{\mathrm{st}}\left(a,b\right)=H_{t}\left(a,b\right)-H_{s}\left(a,b\right)=\frac{1}{4}{\left|a-b\right|}^{2},$$

(4b) $$G_{\mathrm{st}}\left(a,b\right)=\frac{D_{\mathrm{st}}\left(a,b\right)}{H_{t}\left(a,b\right)}\;=\;\frac{{\left|a-b\right|}^{2}}{{4-|a+b|}^{2}},$$

(4c) $$D\left(a,b\right)=\frac{2D_{\mathrm{st}}\left(a,b\right)}{1-H_{s}\left(a,b\right)}\;=\;\frac{{\left|a-b\right|}^{2}}{{\left|a\right|}^{2}+{\left|b\right|}^{2}}.$$

Expressions (4) are equivalent to the (biased) estimators used for calculation of genetic differences as compiled for example in (Jost 2008). Multilocus *D* is calculated as described by (Crawford 2010), and multilocus $G_{\mathrm{ST}}$ is calculated as proposed in (Nei 1973).

### A new concept for estimating directional measures of genetic differentiation

To introduce the new analytic approach, the following hypothetical scenario is considered. Assuming two populations A and B with strong directional gene flow in the direction A → B, but no gene flow in the opposite direction (e.g., as observed when waterfalls prevent or restrict movement in a single direction for species in river systems). In addition, population B may exchange genes with other populations that are genetically distinct from A and thus containing alleles not present in A. How would such a situation be reflected in the allelic frequencies? Generally, it can be expected that most alleles present in population A are also present in B, whereas alleles present in B may or may not be present in A due to the absence of gene flow from B to A.

In the case of neutral loci, the relative allele frequencies of A are expected to be reflected in the migration, and therefore, the proportions of the allele frequencies is assumed to be equal in population B. An idealized example of an allelic matrix (A) is listed in Table 1. In this example, alleles 1 and 2, present in population A, are represented at reduced frequencies but equal proportions in population B, whereas allele 3 is only present in population B. From the distribution of allele frequencies, it becomes evident that there is no gene flow from B to A, but at least some degree of gene flow from A to B cannot be ruled out.

**Table 1:** Allelic matrix *A* of the thought experiment consisting of two populations A and B with directional gene flow from A to B

	Population A	Population B
Allele 1	0.4	0.2
Allele 2	0.6	0.3
Allele 3	0.0	0.5

This concept can be formalized as follows: For each mutual pair of populations to be investigated (populations A and B in this example), a hypothetical pool of migrants with an allelic composition inferred from the two populations surveyed is introduced. That is, the hypothetical population has an allelic distribution ***f***(***a***,***b***). The allelic frequencies represented in the pool are calculated as the (normalized) geometrical means of the allelic frequencies in the corresponding two populations of interest: $f_{i}\left(a,b\right)\;=\;\gamma \sqrt{a_{i}b_{i}}$ with $\gamma \;=\;{\left(\sum_{i}\sqrt{a_{i}b_{i}}\right)}^{-1}$ if $\sum_{i}\sqrt{a_{i}b_{i}}\;>\;0$ and *γ* = 0 otherwise.

Let us present a general motivation of this definition of the hypothetical pool of migrants. From the hypothetical scenario, a number of requirements for the hypothetical pool ***f***(***a***,***b***) are defined:

1. ***f***(***a***,***b***) should be symmetric in its arguments, as the order of populations is arbitrary.
2. Alleles not represented in one of the populations can be assumed not to be relevant for gene flow. As a consequence, ***f*** should be nonzero only for alleles present at nonzero frequencies in both populations.

A general form for ***f***(***a***,***b***) consistent with these two conditions would be

(5) $$f_{i}\left(a,b\right)=\gamma \sum_{k=1}^{K}\gamma_{k}\left(a_{i}^{\alpha_{k}}b_{i}^{\beta_{k}}+a_{i}^{\beta_{k}}b_{i}^{\alpha_{k}}\right)\;\forall i$$

with arbitrary exponents $\alpha_{k},\beta_{k}\;>\;0$ for all *k*, an arbitrary *K* defining the number of summands involved and arbitrary weighting and normalization constants *γ* and $\gamma_{k}$. Here, *arbitrary* means that, at this stage, any function defined through any arbitrary set of the parameters above would be consistent with considerations 1 and 2. In this vein equation (5) defines a quite general class of functions.

In the optimal example data discussed in connection with the hypothetical scenario (Table 1), $a_{1}/a_{2}\;=\;b_{1}/b_{2}$, which mirrors the fact that the proportions of alleles 1 and 2 in population A is reflected in the gene flow and transferred to population B. In order to be consistent with the hypothetical scenario, the pool of migrants is required to reproduce this proportion of alleles as well:

In case of strong gene flow only occurring in one direction between populations, initially with mutually different alleles, $f_{i}\left(a,b\right)/f_{j}\left(a,b\right)\;=\;a_{i}/a_{j}\;=\;b_{i}/b_{j}$ is required for all combination of alleles *i* and *j* present in both populations.
As a vector of allele frequencies, ***f*** needs to be normalized, that is it needs to fulfill $\sum_{i}f_{i}\;=\;1$.

From the 3rd requirement on ***f***, it can be defined that $\alpha_{k}+\beta_{k}\;=\;1$ for any *k*, making an equation of the form

(6) $$f_{i}\left(a,b\right)=\gamma \sum_{k=1}^{K}\gamma_{k}\left(a_{i}^{\alpha_{k}}b_{i}^{1-\alpha_{k}}+a_{i}^{1-\alpha_{k}}b_{i}^{\alpha_{k}}\right)\;\forall i$$

with still arbitrary $0\;<\;\alpha_{k}\;<\;1$ for all *k*, the most general functional approach for the allelic frequencies in the pooled populations. In cases where populations do not share any alleles, ***f*** will not contain any alleles shared with either of these populations. In such cases, genetic differentiation can be set to 1 in both directions.

Equation (6) still reflects a vast class of functions, which could meet our conditions. For the time being, and for the rest of this study, we choose the simplest available function in this class, namely $\alpha_{1}\;=\;\gamma_{1}\;=\;1/2$ and *K* = 1, resulting in

(7) $$f_{i}\left(a,b\right)=\gamma \sqrt{a_{i}b_{i}}\;\forall i$$

with $\gamma \;=\;{\left(\sum_{i}\sqrt{a_{i}b_{i}}\right)}^{-1}$. Hence, the vector of allelic frequencies of the hypothetical population, ***f***(***a***,***b***), is composed of the normalized geometrical means of the respective components of ***a*** and ***b***.

As a measure for directional differentiation, both A and B can now be compared to their shared hypothetical pool of migrants ***f***(***a***,***b***). For this purpose, any standard, nondirectional measures for genetic differentiation can be applied. Here, we choose to use $G_{\mathrm{st}}$ and *D* as introduced in the section Measures of genetic differentiation.

For the general case of a number of *P* populations, a *P* × *P*‐matrix *B* can be defined, containing the directional measures for genetic differentiation, as

(8) $$B\left(A,E\left(\cdot ,\cdot \right)\right)=\left(\begin{array}{ccc} b_{11}\left(A,E\left(\cdot ,\cdot \right)\right) & \cdots & b_{1P}\left(A,E\left(\cdot ,\cdot \right)\right)\\ \vdots & \cdots & \vdots \\ b_{P1}\left(A,E\left(\cdot ,\cdot \right)\right) & \cdots & b_{PP}\left(A,E\left(\cdot ,\cdot \right)\right) \end{array}\right),$$

where the individual elements are defined as

(9) $$b_{ij}\left(A,E\left(\cdot ,\cdot \right)\right)=E\left(a_{i},f\left(a_{i},a_{j}\right)\right)\;\forall i,j.$$

Here, *E*(·,·) is a placeholder for estimates of genetic differentiation, such as $G_{\mathrm{st}}$ and *D*. *A* is the matrix of allele frequencies containing the population‐specific allele frequency vectors ***a***. The value of a particular matrix element $b_{ij}$ can now be taken as an indicator of the potential for gene flow from population *i* to population *j*. Low values indicate a high potential for migration, while high values indicate low potential for migration in the direction of interest.

With the idealized example data listed in Table 1, ***f***(***a***,***b***) = ***a***, implying that the genetic constitution of A is equal to the hypothetical pool of migrants, whereas the genetic differentiation between the pool and population B is nonzero ($G_{\mathrm{st}}\left(b,f\left(a,b\right)\right)\;=\;0.15$ and *D*(***b***,***f***(***a***,***b***)) = 0.42). This result indicates that there is a lower potential for gene flow from B to A than from A to B, which is consistent with the initial hypothetical scenario.

It is important to emphasize that, in this study, we only consider idealized conditions. Thus, the impact of more complex scenarios, including, for instance, recent common ancestry, unequal population sizes, or populations not being at equilibrium, is not considered at this point. The motivation is kept as general and clear as possible to encourage further developments, which allow for adapting the method to more specific and complex conditions. Notwithstanding its simplicity, however, we argue that our model seems to be robust enough to deal with a wide range of conditions.

### Estimations of directional relative migration

Assuming Wright's infinite island model, $F_{\mathrm{st}}$ and by extension $G_{\mathrm{st}}$ can directly be related to migration (Wright 1931).

(10) $$N_{e}m\approx \frac{\left(\left(\frac{1}{G_{st}}\right)-1\right)}{4}$$

Here, we use equation (10) as a step to calculate relative migration; thus, either the infinite or finite island model can be used at this point.

Jost outlines in a similar way how *D* can be used to calculate migration using the finite island model. If we assume that the mutation rate does not differ between populations, *μ* can be eliminated from the Equation (22) in Jost (2008) by defining the scaled migration rate *M* = *m*/*μ*. We fixed the number of sampled populations *n* to two, as we only estimate pairwise comparisons and get

(11) M≈(1−D)/D.

From the effective migration rate in equation (10) and the scaled migration rate in equation (11), relative migration rates between population pairs can be calculated. For now, we only focus on relative migration rates to make the method less sensitive to possible inaccuracies in the estimation of effective migration rate and scaled migration rate, due to populations not fitting the assumptions of the island model or not being in drift–mutation equilibrium. Extending this concept to the directional measures of genetic differentiation introduced in the previous section, a similar expression can be used for the estimation of directional relative migration. To this end, a *P* × *P*‐matrix *C* of relative migration is defined as

(12) $$C\left(A\right)=\left(\begin{array}{ccc} c_{11}\left(A\right) & \cdots & c_{1P}\left(A\right)\\ \vdots & \cdots & \vdots \\ c_{P1}\left(A\right) & \cdots & c_{PP}\left(A\right) \end{array}\right),$$

to calculate migration from $G_{\mathrm{st}}$ the individual elements are defined as

(13) $$c_{ij}\left(A\right)=\frac{\left(\left(\frac{1}{G_{\mathrm{st}}\left(a_{i},f\left(a_{i},a_{j}\right)\right)}\right)-1\right)}{4}\;\forall i,j.$$

to calculate migration from *D* the individual elements are defined as

(14) $$c_{ij}\left(A\right)=\frac{1-D\left(a_{i},f\left(a_{i},a_{j}\right)\right)}{D\left(a_{i},f\left(a_{i},a_{j}\right)\right)}\;\forall i,j.$$

The matrix *C* is then normalized by its largest value to obtain directional relative migration ranging from zero to one. *A* is the matrix of allele frequencies containing the population‐specific allele frequency vectors ***a***. The respective matrix elements $c_{ij}\left(A\right)$ provide an estimate for the directional relative migration from population *i* to population *j*. To assess whether gene flow is significantly asymmetric, the directional genetic distances presented in the previous section can be assessed by means of a bootstrap procedure.

## Simulations

### Methods

To assess the performance of the method described, we evaluated it under variable gene flow patterns and sample designs. This was performed by simulating multiple microsatellite data sets from two populations (*N* = 1000), under three distinct gene flow patterns (*unidirectional gene flow*,* bidirectional symmetric gene flow*, and *bidirectional asymmetric gene flow*). While we acknowledge that these three patterns do not cover all scenarios likely to take place in nature, they are good starting points to begin to assess the potential usefulness of the new method.

The ability of the proposed new approach to resolve each of these migration patterns was tested for three levels of gene flow as follows: low (*m* = 0.00025), medium (*m* = 0.005), and high (*m* = 0.05), which correspond to population divergence levels of $F_{\mathrm{st}}\;=\;0.5,0.05,0.005$ using the equation $F_{\mathrm{st}}\approx \frac{1}{4N_{e}m\;+\;1}$ (Wright 1931).

In cases where the pattern was unidirectional, gene flow was simulated in the direction from population B to population A. For the symmetric bidirectional pattern, gene flow was simulated in both directions at an equal rate (*m*/2). For the asymmetric bidirectional pattern, gene flow was simulated in both directions, but at 1/4 from population A to population B and 3/4 from population B to population A (m*1/4 and m*3/4).

For each combination of gene flow pattern and gene flow rate, 1000 microsatellite data sets were simulated for each sample size starting with *s* = 10 and increasing to *s* = 100 in increments of 10 (i.e., *s* = 10, 20, …, 100). This process was repeated for the number of loci, which were also increased from 10 to 100. Three gene flow patterns, under three levels of migration, tested for 10 different sample sizes and 10 different numbers of loci resulted in 180 unique simulation scenarios, for each of which 1000 simulation replicates were generated. When increasing sample size was evaluated, the number of loci was fixed at 50, and when the number of loci was evaluated, the sample size was fixed at 50.

For each combination of parameters, the number of times out of the 1000 replicates that the method detected higher migration from population B to population A was calculated in percent. For the unidirectional and the asymmetric bidirectional gene flow patterns, the method was expected to detect higher rate in this direction and the result was expected to approach 100%.

In the scenario with symmetric bidirectional gene flow, the method was expected to estimate equally high migration rates in both directions, and therefore, we did not expect to see a signal. In this scenario, due to random chance, half of the simulations where expected to be higher in one direction and the other half to be higher in the other direction. Thus, a value of 50% was the expected outcome.

In all simulations, mutation rate was fixed at $5\times 10^{-4}$, a value that is thought to be common at microsatellite loci in vertebrates (Bhargava and Fuentes, 2010), and base population size was fixed at 2*N* = 2000. The performance of the directional migration method was assessed by calculating relative migration rates derived from both *D* and $G_{\mathrm{st}}$. Relative migration rates were calculated using a newly developed R function, divMigrate from the diveRsity package (Keenan et al. 2013). Simulations were carried out using fastsimcoal2 (Excoffier and Foll 2011), and the whole analysis process was scripted in R (R Core Team 2014). Instructions and R scripts to enable readers to reproduce these analyses are available in a dedicated github repository (https://github.com/lisasundqvist/Sundqvist_et_al_2016).

### Results

#### Unidirectional gene flow

**Figure 1:**
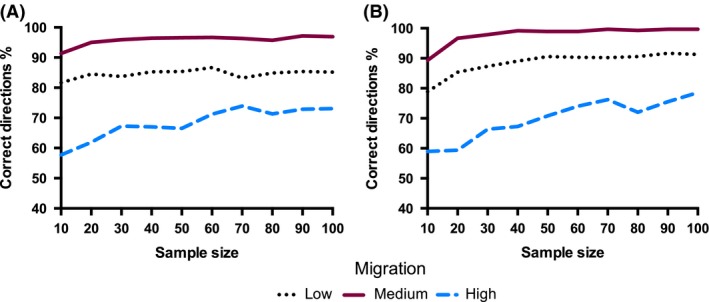
Unidirectional migration: percent correct directions as a function of sample size calculated using *D* (A) and $G_{\mathrm{st}}$ (B). Increasing sample size was evaluated at high (0.05), medium (0.005), and low (0.00025) gene flow. The number of loci was kept fixed at 50.

**Figure 2:**
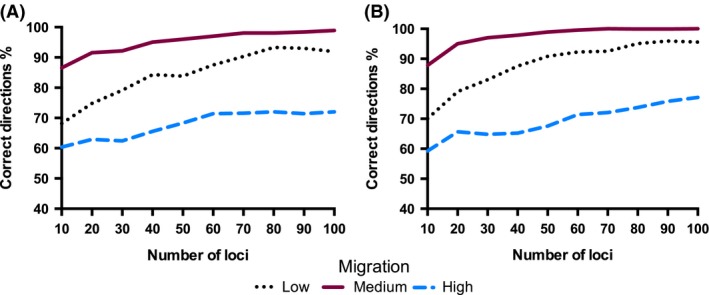
Unidirectional migration: percent correct directions as a function of number of loci calculated using *D* (A) and $G_{\mathrm{st}}$ (B). Increasing number of loci was evaluated at high (0.05), medium (0.005), and low (0.00025) gene flow. The sample size was kept fixed at 50.

The ability of the method to detect the simulated migration from population B to population A was best for the medium migration rate (*m* = 0.005). When the sample size reached 20, the correct direction was found in 95% and more of the simulations for both *D* and $G_{\mathrm{st}}$ (Fig. 1A,B). When the number of loci was increased, the result exceeded 95% when the number of loci was 40 for *D* (Fig. 2A) and 20 for $G_{\mathrm{st}}$ (Fig. 2B). When the number of loci was 60 or higher, the calculations using $G_{\mathrm{st}}$ reached 100% correct directions (Fig. 2B), indicating that the correct direction was detected in all of the 1000 simulations.

The number of correct directions estimated by the method was next highest for low level of migration. For increasing sample size, calculations from $G_{\mathrm{st}}$ reached 90% correct directions when the sample size reached 40. When number of loci varied, the number of correct directions reached 90% for *D* when the number of loci was 70 (Fig. 2A). For $G_{\mathrm{st}}$, the result reached 90% when the number of loci was 50 and 95% when the number of loci reached 80 (Fig. 2B).

When gene flow was high (*m* = 0.05), the least number of correct directions was estimated coming close and up to 75% only for the highest sample size and number of loci tested (Figs 1 and 2). Increasing sample size had highest effect between 10 and 30 (Fig. 1). After that, increase in sample size only improved the results when migration was high.

#### Symmetric bidirectional gene flow

**Figure 3:**
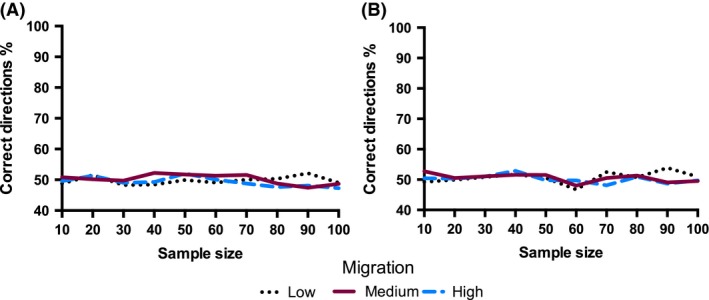
Bidirectional symmetric migration: percent correct directions as a function of sample size calculated using *D* (A) and $G_{\mathrm{st}}$ (B). Increasing sample size was evaluated at high (0.05), medium (0.005), and low (0.00025) gene flow. The number of loci was kept fixed at 50. When migration was symmetric, the expected value was 50%.

**Figure 4:**
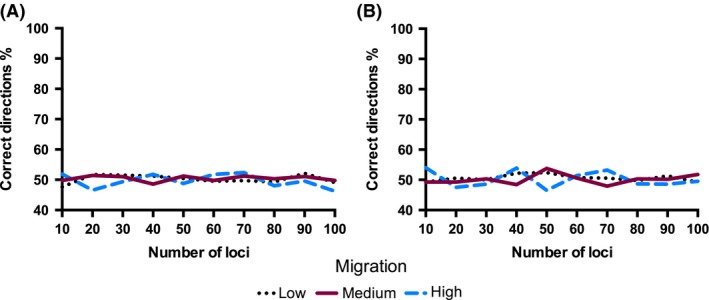
Bidirectional symmetric migration: percent correct directions as a function of number of loci calculated using *D* (A) and $G_{\mathrm{st}}$ (B). Increasing number of loci was evaluated at high (0.05), medium (0.005), and low (0.00025) gene flow. The sample size was kept fixed at 50. When migration was symmetric, the expected value was 50%.

Figures 3 and 4 clearly demonstrate that the method do not systematically show signs of asymmetry when gene flow is symmetric. All values, even when sample size and number of loci was low, are close to the expected value of 50%. When relative migration was calculated from $G_{\mathrm{st}}$ (Figs 3B and 4B), the result was slightly more variable than when calculated from *D* (Figs 3A and 4A).

#### Asymmetric bidirectional gene flow

**Figure 5:**
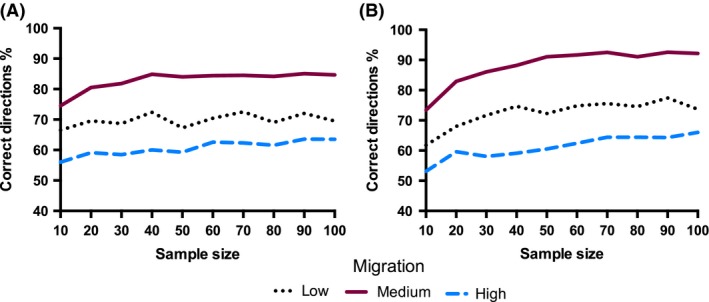
Bidirectional asymmetric migration: percent correct directions as a function of sample size calculated using *D* (A) and $G_{\mathrm{st}}$ (B). Increasing sample size was evaluated at high (0.05), medium (0.005), and low (0.00025) gene flow. The number of loci was kept fixed at 50.

**Figure 6:**
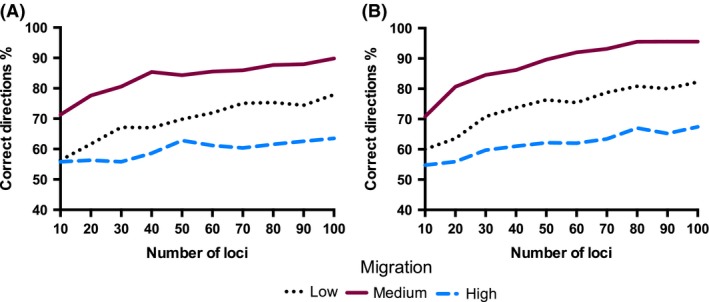
Bidirectional asymmetric migration: percent correct directions as a function of number of loci calculated using *D* (A) and $G_{\mathrm{st}}$ (B). Increasing number of loci was evaluated at high (0.05), medium (0.005), and low (0.00025) gene flow. The sample size was kept fixed at 50.

The usefulness of the method to detect the underlaying migration pattern, which were 1/4 of the migration rate from population A to population B and 3/4 from population B to population A (m*1/4 and m*3/4), is illustrated in Figures 5 and 6. The method performed best under medium migration rate (0.005). The correct direction was then estimated in over 80% of the times for *D* when the sample size was 20 and over (Fig. 5A) and reached 90% for $G_{\mathrm{st}}$ when the sample size exceeded 50 (Fig. 5B). When the number of loci increased, the number of correct directions calculated from *D* reached over 80% when the number of loci was 30 or more (Fig. 6A). For $G_{\mathrm{st}}$, the result reached 80% when the number of loci was 20% and 90% when the number of loci was 50 or more (Fig. 6B).

When the migration rate was low (0.00025), the number of correct directions for estimates calculated from *D* was below 75% for all sample sizes (Fig. 5A). For $G_{\mathrm{st}}$ (Fig. 5B), 75% was reached when the sample size reached 40 and stayed around that value as sample size increased further. When the number of loci increased, 75% was reached when the number of loci was 70 for *D* and 50 for $G_{\mathrm{st}}$, (Fig. 6) and 80% was reached for $G_{\mathrm{st}}$ when the number of loci was 70.

When the migration rate was high (0.05), the method did not estimate the correct direction at a sufficiently high percentage. However, the percentage did increase with sample size and the number of loci (Figs 5 and 6).

**Figure 7:**
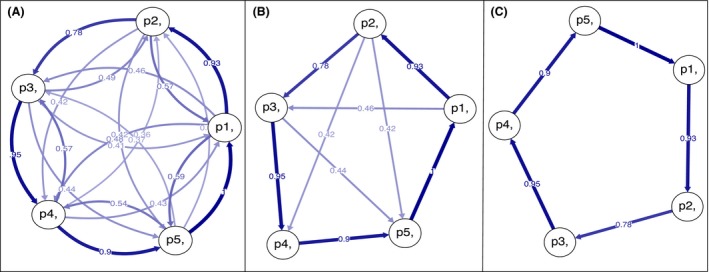
Directional relative migration calculated by divMigrate‐online for the simulated circular stepping stone model with unidirectional migration. (A) Illustrates the calculated migration values. (B) Only includes the values found to be asymmetric, that is they are statistically higher in the shown direction. In (C), the filter threshold for the asymmetric values was set to 0.5.

## Program

To make the new method described in here accessible and easy for people to use, we have developed a web‐based software application called divMigrate‐online. This user‐friendly interface provides integrated network visualizations of gene flow patterns among populations, as well as allowing users to test and visualize significant difference in directional gene flow between pairs of population samples. This section includes a description of the software as well as a demonstration of its use. The flexibility and usefulness of the program are demonstrated by analyzing one simulated data set and one published microsatellite data set of Atlantic salmon (Sandlund et al. 2014b).

### About the program

divMigrate‐online is designed within the shiny framework (RStudio & Inc. 2014) for the R programming language (R Core Team 2014). divMigrate‐online is written in the R and C++ programming languages, integrated using the R package Rcpp (Eddelbuettel et al. 2011) and is hosted at (https://popgen.shinyapps.io/divMigrate-online/).

**Figure 8:**
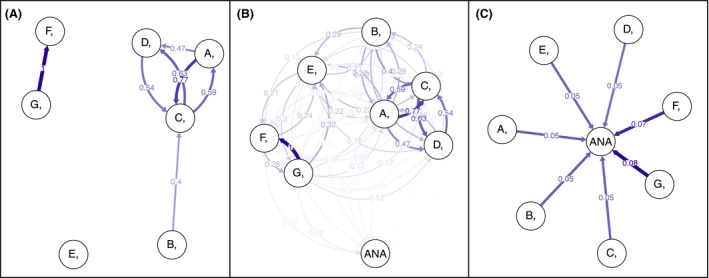
Directional relative migration estimated by divMigrate‐online. (A) Includes population A–G, and the filter threshold was set to 0.35. In (B), data from the anadromous salmon (ANA) are included; no filter was used. (C) Illustrates the migration that was found to be asymmetric; no filter was used.

Estimated gene flow patterns can be visualized and explored using network graphics produced using the qgraph R package (Epskamp et al. 2012). Population samples are represented by nodes in the networks. Each node is hypothetically connected to every other node by two connections, representing the two reciprocal gene flow components between any pair of populations. The properties of these connections, such as their length, shading, and thickness, are determined by the relative strength of gene flow. These properties have a particularly beneficial consequence, namely populations that exchange genes at high rates locally, but low rates elsewhere tend to cluster together within the network space, thus providing a visual illustration of genetic structuring patterns (e.g., Fig. 8A below). DivMigrate‐online also provides an useful filter threshold function that makes it possible to show only values above a specified number. This is a useful feature when getting to know the data set, as it gives the possibility to zoom in and out, especially if many populations are compared simultaneously and the whole picture contains very much information. It can also be useful if low migration rates are not of equal interest as high ones.

The divMigrate‐online application provides an integrated statistical testing facility, where the asymmetry between migration rates is tested. The statistical significance of differences between directional components of gene flow for a population pair is estimated as follows:

1. Resampling of the original data (with replacement) *x* number of times.
2. Estimate allele frequencies from resampled data for both populations as well as their hypothetical pool of migrants
3. Calculate a user‐specified relevant parameter (e.g., *D* or $G_{\mathrm{st}}$) between the hypothetical pool of migrants and both populations for all *x* resamples
4. Construct two 95% confidence intervals of the user‐specified parameter calculated between each population and their shared hypothetical pool of migrants using the quantile method (lower probability = 0.025, upper probability = 0.975).
5. Test for overlap of the estimated 95% confidence intervals. Where there is no overlap, the directional gene flow components are said to be significantly different (asymmetric).

This procedure can be coupled with network visualizations, such that only statistically significant asymmetric links between population pairs are plotted.

While the web application will be sufficient for most users, there may be those who require more flexibility when conducting specialized analyses (e.g., simulation studies where batches of files need to be processed). For this purpose, the methods provided in divMigrate‐online are also implemented in the divMigrate function from the diveRsity R package (Keenan et al. 2013). Users interested in using this function can find out more by typing ?divMigrate into the R console (diveRsity must be installed and loaded first).

### Simulated example

Using fastsimcoal2 (Excoffier and Foll 2011), five populations connected in a circular stepping stone model with unidirectional migration (1→2→3→4→5→1 (rate 0.0005)) was simulated and then analyzed with divMigrate‐online (For details about the simulation, see (https://github.com/lisasundqvist/Sundqvist_et_al_2016)).

Running the data set in divMigrate‐online and choosing *D* to estimate relative migration, the result, as shown in Figure 7A, was generated. All values except very low ones are visualized in this figure. Depending on the amount of data in the data set divMigrate‐online do not always plot all values, all values are, however, included and can be seen in a result matrix. The result in Figure 7A indicates many migration directions that were not simulated; this was not surprising given that the simulated migration pattern was circular and that migration between the populations occurred for many generations. Alleles could thus pass through intermediate populations, and populations not directly connected could experience indirect gene flow.

To further investigate the data set, we asked divMigrate‐online to estimate 1000 bootstrap iterations and calculate confidence intervals for all values to investigate whether the migration was asymmetric. Figure 7B, only shows arrows for the directions that are statistically higher relative to the opposite direction. DivMigrate‐online also provides an useful filter threshold function that makes it possible to filter out low values, thus it is easier to visualize potentially more relevant patterns. In Figure 7C, the filter threshold is set to 0.5 meaning that only the asymmetric values above 0.5 are shown. In this figure, the same migration pattern that was put into the simulation is shown. All network graphics can be exported from the application in various file formats, making the creation of publication standard plots straightforward. It is also possible to download a result matrix including all numbers.

### Empirical example

Recently, Sandlund et al. (2014a) published a study of the spatial and temporal genetic structure of the rare landlocked salmon in a fragmented river in Norway. Using microsatellite genotype data, the authors identified three distinct genetic clusters. The first cluster contained individuals sampled from sites A, B, C, and D, while the second cluster contained individuals sampled from site E, and the third cluster contained individuals sampled from sites F and G. As mentioned previously, the network method used to visualize gene flow patterns in divMigrate‐online has the useful property of representing closely related population samples as local clusters within the network space. To demonstrate this the contemporary microsatellite data from the original study (Sandlund et al. 2014a) were re‐analyzed by divMigrate‐online using Josts *D*. By setting the filter threshold to 0.35, the genetic structure described in (Sandlund et al. 2014a) was reproduced in Figure 8A. Indeed, the groups shown in Figure 8A are similar to the neighbor‐joining dendrogram shown in Figure 4 of (Sandlund et al. 2014a) (excluding the historical samples that were not included in the divMigrate‐online analysis). Further, we included data of the anadromous salmon (ANA) and observed that the landlocked populations were more closely related to each other than to the anadromous salmon. In Figure 8B, the result is shown with no filter. When checking for asymmetric gene flow in the system (Fig. 8C), an interesting result was observed, no significant asymmetry was found between the landlocked populations, but all landlocked populations exhibited asymmetric gene flow to the anadromous salmon population. Considering the history of this system, in particular the fact that these landlocked populations have been isolated from the anadromous salmon for some 9.500 years, it is likely that this pattern is showing us something else. In the definition of this method, we use private alleles or alleles only present in one population as a sign of no migration. In a system that historically has been colonized by a small number of individuals, however, it is likely that the colonized populations share many of its alleles with its source population. It is also likely that the source population displays a higher level of genetic diversity and thus is characterized by the presence of a larger number of private alleles when compared to the colonized population. It is therefore possible that a strong and recent founder effect is the cause of what appears to be asymmetrical migration to the method. This is a particular caveat of the method described in here which potential users should be aware of.

### Accessing the software

divMigrate‐online is a web‐based software application and can be accessed on any operating system through a web browser at https://popgen.shinyapps.io/divMigrate-online/. The application can also be launched locally using R. The procedure is as follows:

1. Install the shiny package in R: install.packages(shiny)
2. Install diveRsity from github (instructions can be found at https://github.com/kkeenan02/diveRsity#development-version
3. Launch divMigrate‐online: shiny::runGitHub(kkeenan02/divMigrateonline)

The source code for the software is freely available at https://github.com/kkeenan02/divMigrate-online. Pull requests can also be made through this repository.

## Discussion

Here, we have introduced a novel alternative approach that estimates directional genetic differentiation and relative migration from any relevant measure of genetic differentiation, thus allowing for the detection of asymmetric gene flow patterns. Tests using simulated data demonstrate that the new approach can detect underlying gene flow patterns with high confidence when migration is present in either one or two directions. The method performed best when gene flow was intermediate (*m* = 0.005). In situations where gene flow was high (*m* = 0.05), the method failed to detect underlying patterns of migration at a sufficiently high percentage for any of the sample sizes or number of loci combinations tested. The low number of times that the simulated direction was found when migration was high is likely linked to the homogenizing effect of high gene flow, resulting in very low differentiation between populations (i.e., $F_{\mathrm{st}}\approx 0.005$). The directional components of gene flow were thus obscured (i.e., the signal to noise ratio was low). However, the upward trend for both increasing sample size and number of loci suggest that under suitable conditions, the method may be capable of resolving such patterns even for cases where genetic structuring is weak.

Based on the simulation results, relative migration calculated from $G_{\mathrm{st}}$ performed slightly better than those calculated from *D*. These results are in line with recent observations of the relative properties of *D* and $G_{\mathrm{st}}$ under variable demographic scenarios (Alcala et al. 2014). Alcala et al. (2014) state that *D* and $G_{\mathrm{st}}$ are complementary measures and suggest general guidelines for when the use of $G_{\mathrm{st}}$ or *D* might be inappropriate. For example, when the expression *θ* < 1 < *nθ* is true (where *θ* is the scaled mutation rate (*θ* = 2*Nμ*) and *n* is the number of populations), as is the case in the simulations used here, the use of $G_{\mathrm{st}}$ is recommended (table 4 in Alcala et al. 2014). Alcala et al. (2014) also introduced a new statistic for the calculation of *Nm* (i.e., the effective number of migrants). This statistic incorporates complementary information from both $G_{\mathrm{st}}$ and *D*, suggesting it may be a more generally suitable measure of migration. When using $Nm_{\mathrm{Alcala}}$ for calculating the percent of correct directions in the different simulation scenarios, the result is very similar to the result obtained when using $G_{\mathrm{st}}$. The simulation results for $Nm_{\mathrm{Alcala}}$ is shown in [Link]. In divMigrate‐online it is possible to calculate directional relative migration from $Nm_{\mathrm{Alcala}}$ as well as from *D* and $G_{\mathrm{st}}$.

The network plots in divMigrate‐online has the very useful property of representing similar population samples as local clusters within the network space. Because the directional migration calculations require that populations are predetermined, this feature is likely to be useful only as a confirmatory complement to more quantitative methods such as those implemented in STRUCTURE (Pritchard et al. 2000), ADEGENET (Jombart 2008), or DAPC (Jombart et al. 2010). The filter threshold function makes it easy to visualize a data set, but how to filter a data set is subjective and it is therefore important to clearly state when and how this function is used.

In certain systems estimates of directional relative migration may be influenced by historic demography, as discussed in the empirical example with the landlocked Salmon. Where historical events such as founder effects or recent common ancestry have influenced the genetic composition of individuals and/or populations, footprints will be left. These footprints may enhance or diminish signs of recent migration in the genetic data. The information about migration is then obscured and makes it difficult for any method to correctly describe the migration patterns. The result of the method can never reveal more information than what is to be found in the allele frequencies examined. Future work might include a more comprehensive exploration of how good the method is to detect signs of founder effects and if it is possible to distinguish them from asymmetric migration. It would also be interesting to investigate how recent common ancestry affects the ability of the method to detect contemporary migration patterns. The composition of populations may also lead to complications. In small populations, for instance, genetic drift plays a bigger role. If a migrating allele is lost due to genetic drift the trace of that migration event will also be lost. If populations are uneven in size the relative impact of an allele will be different in the different populations and it will be difficult to interpret the meaning of the directional relative migration calculated, especially if one of the populations is small. By weighing the allele frequencies of populations proportionally to local size or reproductive values, one could probably improve the results of the method in such situations (Hössjer et al. 2014). In addition, it remains to be examined how robust the method is when applied to system not at equilibrium (Boileau et al. 1992) and with influences of ghost populations (Beerli 2004).

When using allele frequency data, it is possible that alleles present at low frequencies are underestimated due to sampling effects (Gautier et al. 2013; Fung and Keenan 2014). As less common alleles (or alleles only present in one population) are used as an indication of no gene flow, rates estimated by the model may be slightly inflated if applied to imprecisely estimated allele frequencies. However, using alleles only present in one population as an indication of gene flow is a common strategy. Slatkin called these *“private alleles"* and showed that the logarithmic average frequency of private alleles is approximately linearly related to the logarithm of *Nm* (Slatkin 1985). This issue is somewhat dealt with in our model, as its focus is only on relative migration, thus making it possible to assume that the probability of underestimating low frequency alleles is equally high in all populations.

Other available approaches for calculating asymmetric migration, such as Migrate (Beerli 2009) and BayesAss (Wilson and Rannala 2003), while powerful, are known to be difficult to use correctly, due, in part, to the large number of parameters and options that need to be adjusted to the data set under consideration. Thus, the use of these programs as black boxes can lead to misleading results assigned a high confidence (Faubet et al. 2007). Furthermore, these programs are also computationally demanding and, hence, can sometimes take impractical amounts of time to run. In comparison, the method presented here is easy to use, conceptually tangible and computationally efficient.

In conclusion, by introducing the concept of directional genetic differentiation, the novel method presented in here enables users to gain new knowledge about genetic structure in systems that experience asymmetric gene flow. By acquiring such information, it is possible to examine the relationships between the direction of migration to correlated factors such as wind or water currents, which has been performed in Godhe et al. (2013), Sjöqvist et al. (2015) and Godhe et al. (2016). This can lead to new insights about evolutionary processes, as well as allowing for more accurate predictions for the purposes of conservation and management. Of particular note is the utility of the method for understanding metapopulations and their source–sink dynamics. More specifically, the ability to identify low‐quality sink populations as well as high‐quality source populations is a major challenge in conservation genetics and is readily possible using the method presented here.

## Conflict of Interest

None declared.

## Data Accessibility

Only simulated and already published data is used in this manuscript, computer code is freely available at github repository (https://github.com/lisasundqvist/Sundqvist_et_al_2016).

## Figures and Tables

**Figure A1:**
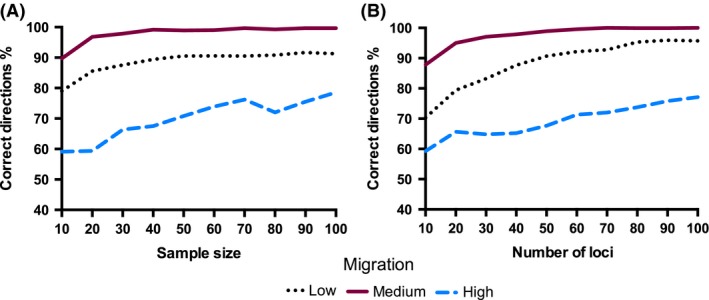
Unidirectional migration: percent correct directions as a function of sample size (A) and number of loci (B) calculated using $Nm_{\mathrm{Alcala}}$. Increasing sample size and number of loci were evaluated at high (0.05), medium (0.005), and low (0.00025) gene flow. When sample size was evaluated, the number of loci was kept fixed at 50, and when the number of loci was evaluated, the sample size was kept fixed at 50.

**Figure A2:**
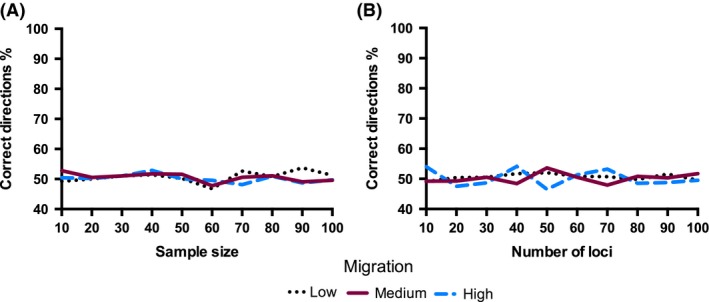
Bidirectional symmetric migration: percent correct directions as a function of sample size (A) and number of loci (B) calculated using $Nm_{\mathrm{Alcala}}$. Increasing sample size and number of loci were evaluated at high (0.05), medium (0.005), and low (0.00025) gene flow. When sample size was evaluated, the number of loci was kept fixed at 50 and when the number of loci was evaluated the sample size was kept fixed at 50. When migration was symmetric, the expected value was 50%.

**Figure A3:**
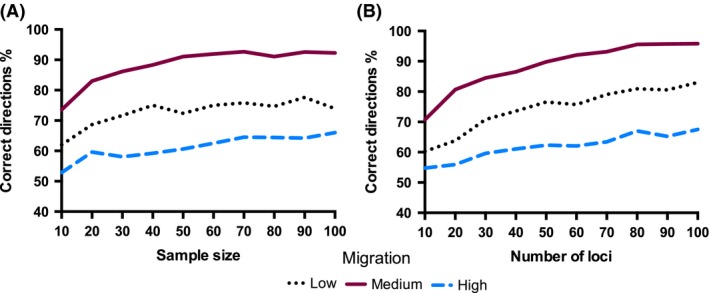
Bidirectional asymmetric migration: percent correct directions as a function of sample size (A) and number of loci (B) calculated using $Nm_{\mathrm{Alcala}}$. Increasing sample size and number of loci were evaluated at high (0.05), medium (0.005), and low (0.00025) gene flow. When sample size was evaluated, the number of loci was kept fixed at 50, and when the number of loci was evaluated, the sample size was kept fixed at 50.
